# Draft Genome Sequences of *Escherichia coli* O157:H7 Strains Rafaela_II (Clade 8) and 7.1_Anguil (Clade 6) from Cattle in Argentina

**DOI:** 10.1128/genomeA.00617-15

**Published:** 2015-06-11

**Authors:** Ariel Fernando Amadio, Natalia Amigo, Andrea Fabiana Puebla, Marisa Diana Farber, Angel Adrián Cataldi

**Affiliations:** a Estación Experimental Agropecuaria Rafaela, Instituto Nacional de Tecnología Agropecuaria (INTA), Rafaela, Santa Fe, Argentina; b Centro de Investigación en Ciencias Veterinarias y Agronómicas (CICVyA), Instituto de Biotecnología, Instituto Nacional de Tecnología Agropecuaria (INTA), Hurlingham, Buenos Aires, Argentina; c Consejo Nacional de Investigaciones Científicas y Técnicas (CONICET), Buenos Aires, Argentina

## Abstract

*Escherichia coli* O157:H7 is a major etiologic agent of diseases in humans that cause diarrhea, hemorrhagic colitis, and hemolytic-uremic syndrome. Here, we report the draft genome sequences of two strains isolated from cattle that had high levels of Shiga toxin 2 and high lethality in mice.

## GENOME ANNOUNCEMENT

*Escherichia coli* O157:H7 is the etiologic agent of hemolytic-uremic syndrome. This is the leading cause of chronic renal failure in children in Argentina and several other countries (1). This bacterium produces Shiga toxin (Stx) types 1 and 2 (2, 3), which are responsible for systemic damage. The main reservoir of *E. coli* O157:H7 is cattle, which harbor this organism in their intestinal tract (4, 5), especially on the lymphoid follicle-dense mucosa at the terminal rectum (6). Fecal contamination of meat during slaughter, the use of feces as fertilizer, and the contamination of drinking water (5, 7) cause the entry of these bacteria into the human food chain. Single nucleotide polymorphism (SNP) typing was previously used to define nine *E. coli* O157:H7 clades, and the clade 8 strains were found to be more associated with severe disease (8).

This work reports the draft genome sequences of two *E. coli* O157:H7 strains isolated from cattle in the central humid Pampas, Argentina, in 2009. Strain Rafaela_II belongs to clade 8, and strain 7.1_Anguil belongs to clade 6. Both strains produced elevated levels of Shiga toxin 2 and had high lethality in mice (N. Amigo, E. Mercado, A. Bentancor, P. Singh, D. Vilte, E. Gerhardt, E. Zotta, C. Ibarra, S. D. Manning, M. Larzábal, and A. Cataldi, unpublished data).

Genomic DNA was isolated using a standard chloroform-isoamilic alcohol extraction. Paired-end Nextera XT libraries were constructed (500-bp insert size) and sequenced in an Illumina MiSeq sequencer (2 × 250 bp). Raw sequences were quality trimmed with Sickle (9), resulting in 1,262,211 and 1,048,969 sequences for 7.1_Anguil and Rafaela_II, respectively. *De novo* assembly was done using SPAdes version 3.1.0 (10). For 7.1_Anguil, 244 scaffolds >500 bp were obtained, the largest being 375,850 bp, with an *N*_50_ of 146,432 bp and a G+C content of 50.46%. For Rafaela_II, 232 scaffolds >500 bp were obtained, the largest being 375,042 bp, with an *N*_50_ of 147,588 bp and a G+C content of 50.28%. Scaffolds were ordered using ABACAS (11) and compared with the genomes of *E. coli* O157:H7 strains Tw14359 (12) and EDL933 (13) using BLAST and ACT (14) to analyze structural differences. Variants at the nucleotide level were analyzed using *breseq* (15) and the same references. Both strains carry the large virulence plasmid pO157, but strain 7.1_Anguil has an additional plasmid highly homologous to avian pathogenic *E. coli* (APEC) strain 7122 (O78:K80:H9) plasmid pChi7122-3.

Genes were predicted with Prodigal version 2.6.1 (16), obtaining 5,643 and 5,439 genes for 7.1_Anguil and Rafaela_II, respectively. Using GET_HOMOLOGUES (17), we compared the gene content using the OrthoMCL methodology (18) to obtain shared and unique genes using strains TW14359 and EDL933 as references. The comparison of shared genes showed a closer phylogenetic relationship between isolate 7.1_Anguil and reference strain EDL933, while isolate Rafaela_II is closely related to TW14359. This is also supported by a previously performed SNP analysis (15). Based on core and pangenome analysis, a considerable number of exclusive genes from Rafaela_II (272) and 7.1_Anguil (447) are under analysis.

### Nucleotide sequence accession numbers. 

These whole-genome shotgun (WGS) projects have been deposited at DDBJ/EMBL/GenBank under the accession numbers LAZD00000000 for 7.1_Anguil and LAYW00000000 for Rafaela_II. The versions described in this paper are LAZD01000000 and LAYW01000000, respectively.
